# Single-feature polymorphism mapping of isogenic rice lines identifies the influence of terpene synthase on brown planthopper feeding preferences

**DOI:** 10.1186/1939-8433-6-18

**Published:** 2013-08-02

**Authors:** Wintai Kamolsukyunyong, Wissarut Sukhaket, Vinitchan Ruanjaichon, Theerayut Toojinda, Apichart Vanavichit

**Affiliations:** Rice Gene Discovery Unit, National Center for Genetic Engineering and Biotechnology (BIOTEC), Kasetsart University, Kamphaeng Saen, Nakhon Pathom 73140 Thailand; Interdisciplinary Graduate Program in Genetic Engineering, Kasetsart University, Chatuchak, Bangkok, 10900 Thailand; Rice Science Center, Kasetsart University, Kamphaeng Saen, Nakhon Pathom 73140 Thailand; Agronomy Department, Faculty of Agriculture, Kasetsart University, Kamphaeng Saen, Nakhon Pathom 73140 Thailand

**Keywords:** Brown planthopper, Single-feature polymorphism (SFP), Sesquiterpene synthase (STPS), Antixenosis on feeding preference (AFP)

## Abstract

**Background:**

*Bph3*, a major brown planthopper (BPH) resistance locus derived from the rice cultivar Rathu Heenati (RH), has been used as a stable donor of traits that improve highly susceptible aromatic rice varieties in Thailand. Map-based cloning was initiated using a set of isogenic lines (ILs) harboring the major *Bph3* locus on chromosome 6. IL genomes were scanned with a 57 K Affymetrix Rice GeneChip to identify the gene responsible for *Bph3*.

**Findings:**

Single-feature polymorphism (SFP) mapping was used to localize 84 candidate genes. An expression analysis of 15 selected candidate genes in the aromatic rice cultivar KDML105 (KD) and the ILs under normal conditions revealed two differentially expressed sequences. Following hopper feeding, only one candidate gene, Os04g27430, was differentially expressed. Os04g27430 encodes a putative *sesquiterpene synthase* (*STPS*) gene that was induced by BPH feeding in ILs. An antixenosis test in three selected ILs revealed a major role for *STPS* in insect preference during the first 120 hours of the rice-insect interaction. Functional SNPs in exon 5 that resulted in the deletion of seven amino acids in the susceptible rice line were identified. Moreover, three additional SNPs associated with three transcription binding sites were also identified, which might explain the differential response of Os04g27430 during the anti-feeding test.

**Conclusion:**

Os04g27430 is the second known rice *STPS* induced by BPH. The gene may involve an antixenosis BPH resistance mechanism. The combination of the *STPS* and the *Bph3* locus was more effective than *Bph3* alone in the tested ILs.

**Electronic supplementary material:**

The online version of this article (doi:10.1186/1939-8433-6-18) contains supplementary material, which is available to authorized users.

## Findings

### Microarray-based genome mapping identification of additional genes correlated with brown planthopper (BPH) resistance in rice

**Figure 1:**
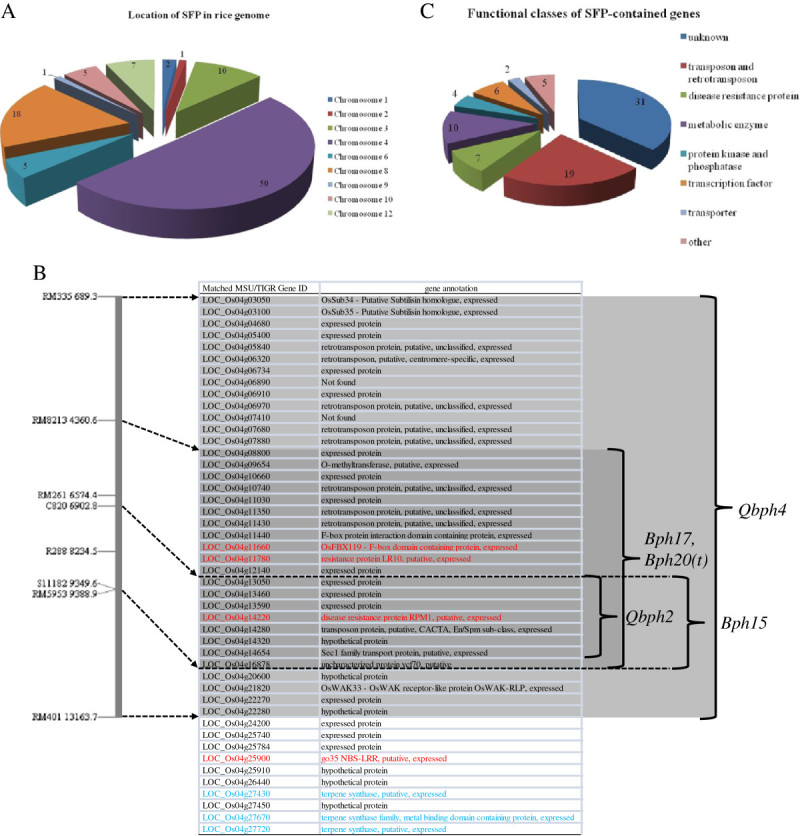
**Classification of SFP and SFP-containing genes. (A)** Pie chart depicting the chromosomal location of 99 predicted unique SFPs. **(B)** SFP-containing genes located on chromosome 4 and the location of BPH resistance genes mapped to chromosome 4. The vertical bar represents the map location on chromosome 4. RM markers and their physical location on the pseudomolecule are shown at the left. The SFP-containing genes located in the *Qbph2*, *Qbph4*, *Bph15*, and *Bph17* regions are highlighted. The three SFP-containing *TPS* genes are labeled in blue. The SFP-containing resistance related genes are labeled in red. **(C)** Pie chart showing the functional classification of 84 SFP-containing genes. Numeric characters in the pie charts indicate the number of SFPs or genes located or classified in that chromosome/class.

The stability of brown planthopper (BPH) resistance in Rathu Heenati (RH), a traditional Sri Lankan rice cultivar containing *BPH 3*, has made this strain one of the most popular hopper resistance donors in the Mekong subregion, where rice production is highly intensive. The Thai jasmine rice KDML105 (KD) is one of the most sensitive cultivars to BPH, and its BPH resistance has recently been improved by backcross introgression of the critical *Bph3* region linked to RM589 on chromosome 6 from RH (Jairin et al. 2009). BC_3_F_5_ isogenic lines (ILs), differing primarily in the introgressed region, were developed. The Affymetrix Rice GeneChip array was used to scrutinize the critical map region on chromosome 6 to simplify the map-based cloning of *BPH 3*. The pool of genomic DNA from four ILs with commonly inherited BPH resistance from RH – UBN3078-101-342-4-162 (IL162), UBN3078-101-342-4-283 (IL283), UBN3078-101-342-6-302 (IL302), and UBN3078-101-432-6-308 (IL308) – was used for single-feature polymorphism (SFP) mapping comparing the genomic DNA of KD. SFP mapping was performed following the protocol developed by Kumar et al. 2007. No significant variation between samples or replicates was observed. A statistical analysis of the microarrays was performed, and SFPs were predicted by determining the hybridization differences at each perfect match (PM) probe in the array (Thongjuea et al. 2009). At a false-discovery rate of 30%, 157 PM probes were selected for further investigation (Additional file 1). Only 99 SFPs had unique locations in the rice nuclear genome; the remaining 58 SFPs were located in multiple locations, no locations, or in organellar genomes. The 99 SFPs were located on 84 annotated genes throughout the rice genome. Five SFPs were located on chromosome 6 and were all outside the critically mapped location (Figure 1A) (Jairin et al. 2007). This observation was consistent with the expression analysis of BPH-infested RH conducted by another research group that did not identify any gene in the *Bph3* candidate region (Wang et al. 2012). By contrast, half of the SFPs were located on chromosome 4 (Additional file 2: Table S1), where another BPH resistance gene from RH (*Bph17*) as well as other BPH resistance genes (*Bph12(t)*, *Bph15, Bph20(t)*, *Qbph2*, and *Qbph4*) have been previously mapped (Sun et al. 2005 Yang et al 2002,2004 Rahman et al. 2009 Huang et al. 2001 Liu et al. 2009).

*Qbph4, Bph17*, and *Bph20(t)* were mapped to the intervals RM335 – RM401 and RM8213 – RM5953 and with the linked marker RM5953, respectively. The *Qbph4* region encompassed a position from 689,354 to 13,163,724 bp, and *Bph17* encompassed 4,360,621 to 9,388,937 bp on pseudomolecule 4 (Os-*Nipponbare*-Reference-IRGSP-1.0). A total of 36 SFP-containing genes (Os04g03050 – Os04g22280) were located in the *Qbph4* region (Figure 1B). Nineteen of these genes (Os04g08800 – Os04g16878) were also specifically associated with the *Bph17* region. The *Qbph2* and *Bph15* genes were mapped in the same region by the RFLP markers C820-R288 and C820-S11182. The region located between 6,902,846 and 9,349,627 bp on pseudomolecule 4 contained eight SFP-containing genes (Os04g13050 – Os04g16878). The linked marker of *Bph12(t)*, RM261, was adjacent to Os04g11780, the resistance protein LR10, with a physical distance of 130.5 kb. Moreover, another NBS-LRR resistance-related protein, Os04g25900, also contained an SFP.

Compared to the expression analysis of BPH-infested RH studied by Wang et al. (2012), three BPH resistance-related genes, two putative resistance proteins, Os04g11780 (LR10) and Os04g14220 (RPM1), and an F-box-containing protein, Os04g11660*,* were found to contain SFP (Figure 1B and Additional file 2: Table S1) in the present study. In contrast, no candidate BPH resistance gene on chromosome 3, 6, and 10 identified in the study by Wang et al. (2012) was found to contain an SFP in our study. This difference may be due to the different BPH-susceptible rice cultivars, KD and Taichung native 1 (TN1), used for the RH comparison in the two studies.

The SFP-containing genes were classified into various functional groups, as shown in Figure 1C. The largest group contained genes with unknown functions such as expressed proteins, hypothetical proteins, and uncharacterized proteins (Additional file 3: Table S2). Transposons and retrotransposons formed the second largest group. The most significant finding was the identification of 10 genes that encode metabolic enzymes in the third most abundant group, which included three genes encoding terpene synthases (TPS). These enzymes are involved in the biosynthesis of secondary metabolites known as terpenoids, a large group of volatile compounds involved in defense mechanisms against plant herbivores (Schnee et al. 2006 Yuan et al. 2008). The fourth most abundant group included seven R gene-like sequences on chromosomes 3, 4, 8, and 10. These findings suggest that several minor quantitative trait loci (QTLs) may strengthen *BPH 3* in terms of stable BPH resistance in RH and ILs. The last three groups contained genes involved in protein phosphorylation processes, transcription factors, and transporters.

### Expression analysis of SFP-containing genes

**Figure 2:**
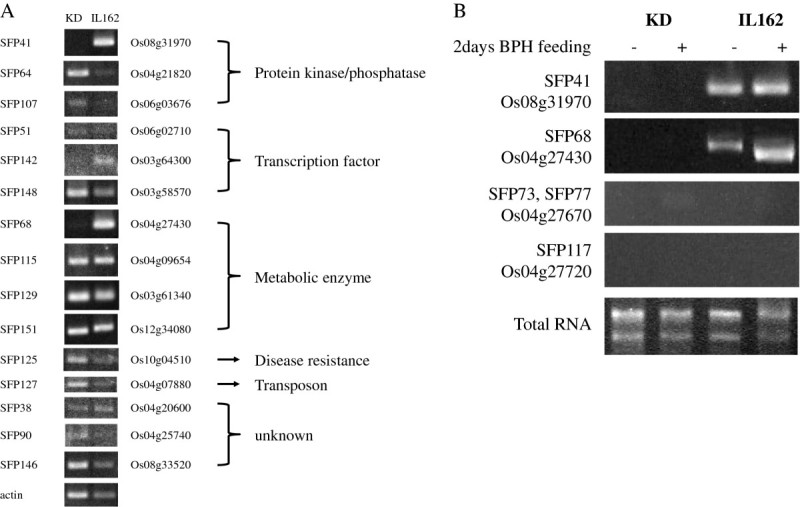
**Expression analysis of SFP-containing genes by RT-PCR. (A)** RT-PCR analysis of rice lines KD and IL162. SFP and LOC numbers are from (Additional file 2: Table S1). Actin was used as a positive control. The functional classes of these genes refer to the functional classes in (Additional file 3: Table S2). **(B)** Expression analysis of rice plants following two days of BPH infestation (+) and control plants (−). The total RNA in each lane is represented by the amount of RNA used in the reaction.

A total of 87 predicted SFPs were validated by comparing the hybridization intensity of each probe with the results of sequence comparisons and PCR amplifications (Additional file 4). A total of 15 genes were chosen from 24 validated SFP-containing genes in which an SNP or a small insertion/deletion between KD and RH was identified (Additional file 5: Table S3). Reverse transcription polymerase chain reaction (RT-PCR) expression analyses and the functional ontologies of 15 pre-candidate genes are shown (Figure 2A). The Os08g31970 and Os04g27430 genes clearly exhibited differential expression under normal conditions, with no expression in the susceptible KD jasmine rice. In contrast, several pre-candidate genes were differentially expressed in the susceptible parent; however, the remaining genes exhibited constitutive expression. The differential expression of Os08g31970 and Os04g27430, an NHL repeat-containing protein that plays a role in signal transduction and a TPS responsible for the biosynthesis of volatile compounds, respectively, was further verified. Os04g27670 and Os04g27720 (two SFP-containing *TPS* genes, Figure 1B) were also selected for a total of four genes that were evaluated in a two-day BPH feeding test using two-week-old seedlings and the Ubon Ratchathani biotype of the BPH population (UBN-BPH) (Jairin et al. 2009). Interestingly, only Os04g27430 exhibited differential expression between the control and the BPH feeding condition in IL162; however, no change in expression levels was observed for Os08g31970 in IL162 (Figure 2B). No expression was detected for the remaining two *TPS* genes in the rice plants, perhaps because the genes were not functional during the seedling stage or because they may be pseudogenes. We investigated the role of Os04g27430 in response to BPH attack in greater detail.

### Genomic characterization of Os04g27430

**Figure 3:**
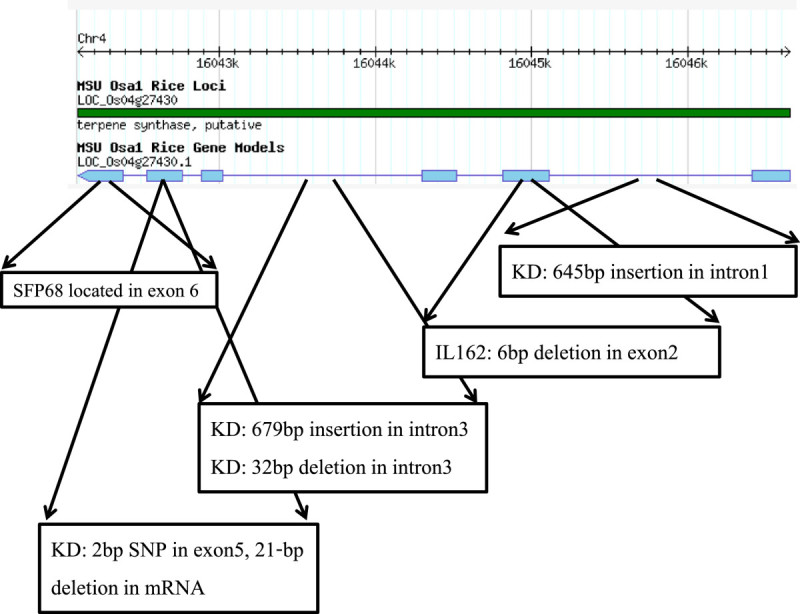
**Gene construct of Os04g27430 from the Rice Genome Annotation Database** (http://rice.plantbiology.msu.edu)**.** Major polymorphisms between the KD and RH alleles (SNP or insertion/ deletion) are marked in horizontal boxes below the construct.

**Figure 4:**
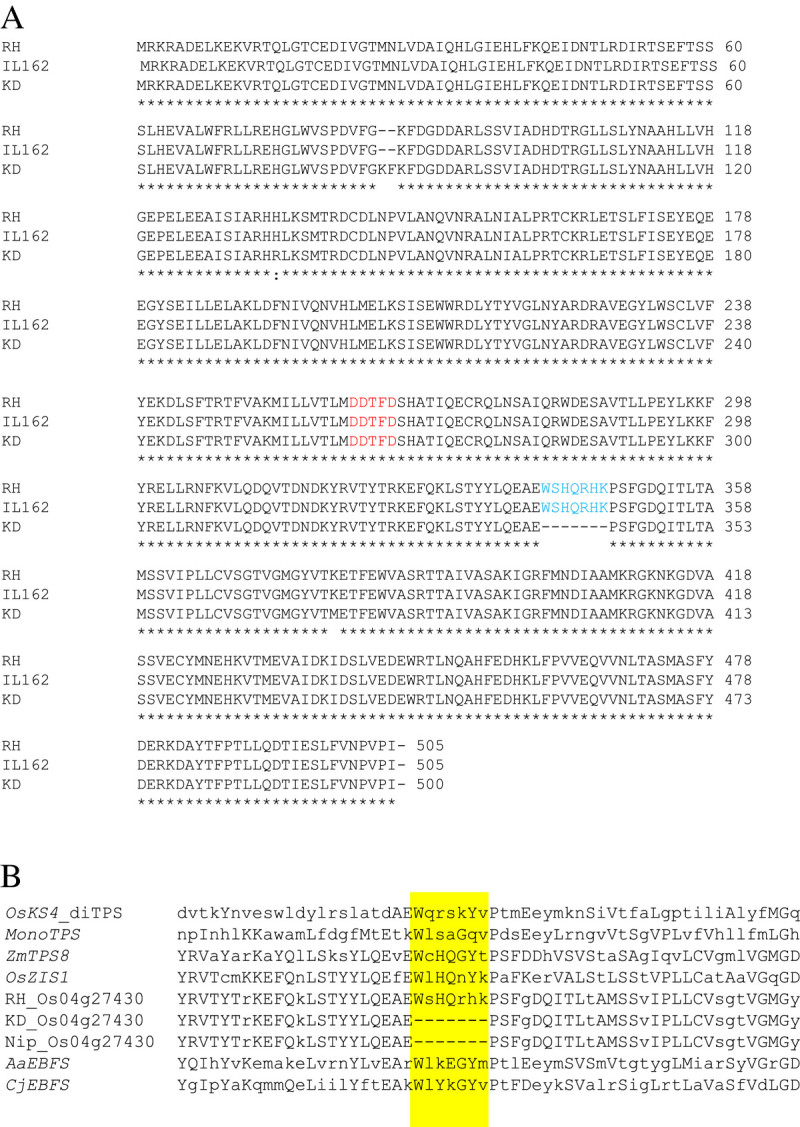
**Amino acid sequence analysis. (A)** Amino acid sequence alignment of the KD, RH, and IL162 alleles of Os04g27430. The conserved DDXXD domain found in the plant *TPS* genes is labeled in red, and the seven-amino-acid deletion in the KD allele is labeled in blue. **(B)** Consensus sequence detected in Os04g27430 and the *TPS* genes of rice and other plant species. *OsKS4* and *monoTPS* are rice *TPS* s, *ZmTPS8* is a terpene synthase from *Zea mays* (NP_001105912), *OsZIS1* is a rice putative *zingiberene synthase I* (ACM41835), *AaEBFS* is the *(E)-β-farnesene synthase* from *Artemisia annua* (AAX39387), and *CjEBFS* is the *(E)-β-farnesene synthase* from *Citrus junos* (AAK54279).

The genomic region of Os04g27430 in RH, KD, and IL162 was sequenced and compared (accession nos. KC511049 – KC511051), and major polymorphisms were identified (Figure 3). Three indels were found to be located in intron 1 (645 bp) and intron 3 (679 and 32 bp, respectively). In the coding sequence, an in-frame 6-bp deletion was present in exon 2 in RH and IL162. More significantly, a 2-bp SNP was present in KD exon 5 with a strong correlation to a 21-bp deletion in its cDNA. Comparisons between the genomic DNA, cDNA, and predicted amino acid sequences (accession nos. KC511027 – KC511029) revealed a 21-bp deletion in exon 5 of the KD cDNA allele that resulted in the deletion of seven residues from the amino acid sequence in KD. The deduced amino acid sequences of the KD and RH alleles were translated, yielding protein sequences containing 500 and 505 residues, respectively. The conserved DDXXD motif that functions as a substrate binding site was present in both the KD and RH alleles (Figure 4A). The seven-amino-acid deletion was WxHQxxx, a signature motif in several plant *TPS* genes (Figure 4B). Based on the presence of this important deletion in KD, Os04g27430 mRNA may be subjected to post-transcriptional degradation, and the absence of this mRNA may responsible for the BPH susceptibility observed in the KD parent.

**Figure 5:**
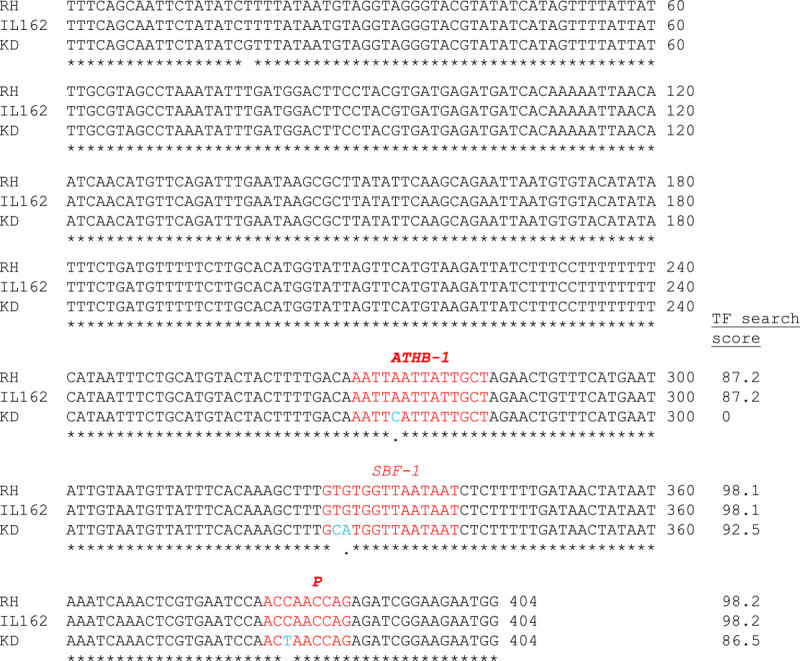
**Genomic sequence alignment of the 5′ region of Os04g27430.** The 3 TF elements are marked in red, and the SNPs of the KD allele are marked in blue. The TF search score for each allele is indicated at the far right of the alignment.

The 5′ upstream region of Os04g27430 in RH, KD, and IL162 was sequenced (accession nos. KC511031, KC527594, KC511035), and searches for transcription factor (TF) binding sites were performed (http://www.cbrc.jp/research/db/TFSEARCH.html). Three consensus elements for the transcription factors *ATHB-1* (*Arabidopsis thaliana* homeobox protein 1), *SBF-1* (silencer-binding factor 1), and *P* (maize activator P) were identified in this promoter region. Interestingly, the KD allele contained one SNP in each element. These SNPs led to the non-recognition of the *ATHB-1* element (score = 0) and a decreased TF search score for the *SBF-1* and *P* elements (scores = 92.5 and 86.5) (Figure 5). These three SNPs, particularly the SNP in the *ATHB-1* element, may be responsible for the low expression levels of this gene in KD because the transcription factor cannot bind at the target site to enhance gene expression. Therefore, a sequence comparison of other BPH-resistant rice varieties is needed to further explore the understanding of how this gene is controlled.

**Figure 6:**
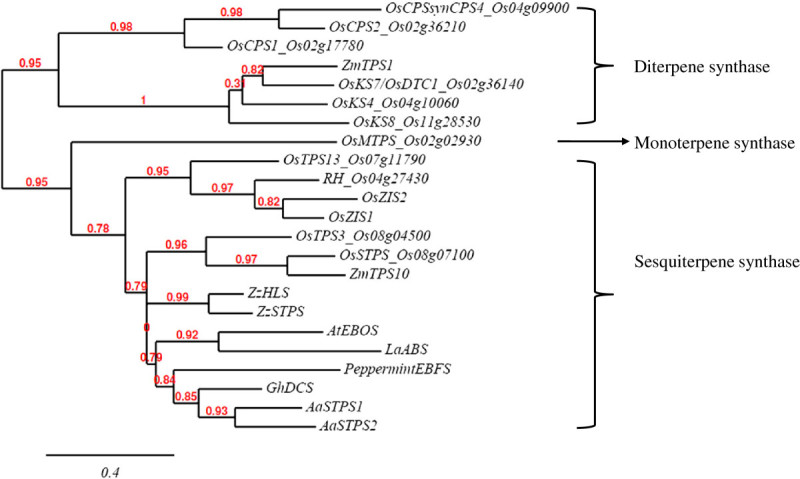
**Phylogenetic analysis based on the degree of sequence similarity between Os04g27430 and other rice TPS genes.** Rice gene identities are based on the gene name in the reference and the rice genome database; the prefix ‘LOC_’ is omitted. *ZmTPS1* and *ZmTPS10* are *TPS* from *Zea mays* (AAO18435, AAX99146), *AaSTPS1* and *2* are *sesquiterpene cyclases* from *Artemisia annua* (CAC12732, AAG24640), *OsZIS1* and *2* are rice putative *zingiberene synthases* (ACM41835, ACM41834), *ZzAHS* and *ZzSTPS* are *α-humulene synthase* and *sesquiterpene synthase* 4 from *Zingiber zerumbet* (BAG12020, BAG50434), PeppermintEBFS is *(E)-β-farnesene synthase* from peppermint tree (Mentha x piperita) (AAB95209), *GhDCS* is a *δ-cadinene synthase* from *Gossypium hirsutum* (AF270425), *AtEBOS* is *(E)-β-ocimene synthase* from *Arabidopsis thaliana* (NP_567511.3), and *LaABS* is *α-bergamotene synthase* from *Lavandula angustifolia* (ABB73046).

The amino acid sequences of 10 rice *TPS* s (Cheng et al. 2007 Prisic et al. 2004 Sakamoto et al. 2004 Xu et al. 2004 Yuan et al. 2008) and *TPS* s from other plant species were included in the phylogenetic analysis of the RH allele of Os04g27430, which revealed that Os04g27430 clustered with the *sesquiterpene synthase* (*STPS*) group (Figure 6). Os04g27430 was most similar to *OsZIS1*, *OsZIS2*, and *OsTPS13*. *OsZIS1* and *2* are putative *zingiberene synthase* genes whose function has not been confirmed experimentally.

**Figure 7:**
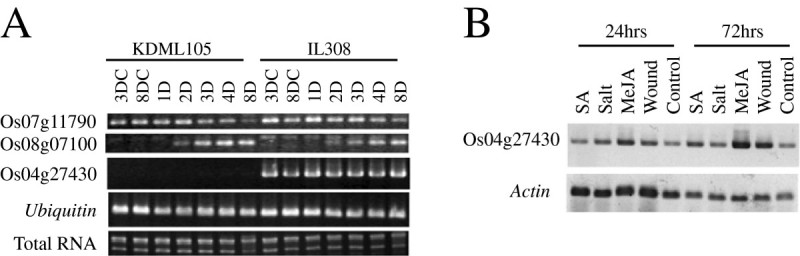
**Expression analysis of** ***STPS*** **genes. (A)** The expression of three *STPS* s in KD and IL308 under control (3DC and 8DC) and BPH feeding (1D, 2D, 3D, 4D, and 8D) conditions; D = day(s). *Ubiquitin* and total RNA were used as positive controls for RT-PCR. **(B)** Expression of Os04g27430 in response to stress conditions by RT-PCR analysis. *Actin* was used as a positive control. Two-week-old RH seedlings were subjected to several stresses. For SA and MeJA, the seedlings were sprayed with 1 mM and 250 μM salicylic acid and methyl jasmonate, respectively; for the salt treatment, the seedlings were transplanted into 150 mM NaCl solution; and for wounding, the seedlings were cut into small pieces in distilled water.

*OsTPS13* is an *STPS* that catalyzes the formation of the sesquiterpene alcohol (E, E) farnesol (Cheng et al. 2007). The gene was identified from methyl jasmonate (MeJA)-treated rice seedlings. However, this gene was constitutively expressed in two-week-old KD and IL308 seedlings under both control and BPH feeding conditions for 1, 2, 3, 4, and 8 days (Figure 7A). In addition to BPH feeding, 24 and 72 hr of MeJA and wound stress also induced Os04g27430 expression (Figure 7B). This discovery suggests that *OsTPS13* is not a BPH feeding-inducible *STPS* and that Os04g27430 expression is induced by both BPH feeding and other stresses, such as MeJA and wounding.

Another *STPS* gene (Os08g07100) that is reportedly induced by BPH (Cho et al. 2005) was not polymorphic between KD and IL308 at the expression level in the present study (Figure 7A). This gene was induced by BPH feeding in both rice strains. Moreover, the gene was induced by BPH feeding and by the fall army worm (Yuan et al. 2008), suggesting that the gene plays a common role in the response to herbivore attacks on rice plants.

Os04g27430 is likely the *STPS* that functions as *zingiberene synthase*, which catalyzes the formation of a number of sesquiterpene products (Iijima et al. 2004). Sesquiterpene volatile compounds are the potential products of this gene and may play a role in BPH resistance mechanisms in RH and ILs.

Allelic variation in *TPS* genes leading to a volatile compound composition difference has been reported in maize (Köllner et al. 2004). In rice, several rice varieties have been shown to release different volatile blends (Lou et al. 2006); however, the gene(s) that controls these differences has not yet been identified. In our preliminary study, a total of 25 sesquiterpenes were identified by GC-MS in the mixture of volatile compounds emitted by KD and RH rice plants infested by BPH. The major sesquiterpene product that KD emitted at a significantly lower level than RH is E-β-farnesene (data not shown), the common constituent of the aphid alarm pheromone (Bowers et al. 1972 Edwards et al. 1973 Pickett and Griffiths. 1980) and the aphid repellent of wild potato (Gibson and Pickett. 1983). This variation may be due to a defect in the KD allele of Os04g27430 at both the genomic and the protein sequence levels.

### Os04g27430 may play a more important role than Bph3 in the antixenosis mechanism

**Figure 8:**
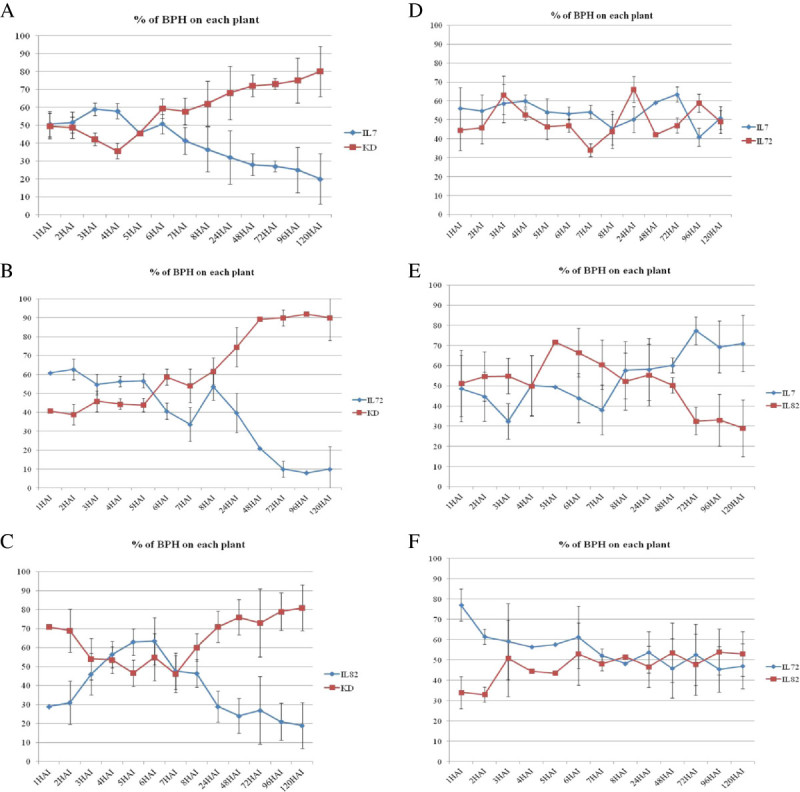
**Antixenosis of BPH feeding preference (AFP) test. (A)**-**(C)** show comparisons between KD and the ILs; **(D)**-**(F)** provide comparison between the ILs. A total of 10 second- and third-instar BPH nymphs were allowed to settle on the experimental plants, and eight replicates were performed for each comparison. The y-axis indicates the number of BPH that settled on each plant as a percentage, and the x-axis shows the progress between 1 and 120 hr after BPH infestation.

Three ILs were selected to elucidate the epistasis of Os04g27430 and the *BPH 3* major gene within the BPH resistance mechanism. UBN03078-101-342-4-7(IL7), UBN03078-101-342-4-72 (IL72), and UBN03078-101-342-6-82 (IL82) contain a *Bph3* critical region, Os04g27430, and both, respectively. The antixenosis of the BPH feeding preference (AFP) was compared between each IL with KD. Within the first 8 hr, UNB-BPHs randomly landed on each tested plant and consistently moved to KD. At 120 hr, the majority of the UBN-BPH population (> 80%) had settled on KD instead of the IL plants (Figure 8A-8C). This result as well as an AFP test between IL7 and IL72 suggest that either *Bph3* or Os04g27430 is sufficient to confer protection against BPH landing within the first 120 hr of attack (Figure 8D). In a comparison between IL82, which contains both *Bph3* and Os04g27430, with IL7 and IL72 (Figure 8E-8F), UBN-BPH was found to prefer IL7 over both IL72 and IL82. This result suggests that Os04g27430 is more important than *Bph3* in determining BPH landing preference.

This study clearly demonstrates that the *Bph3* critical region and at least 84 other genes have been transferred to ILs. This unexpected finding may be a consequence of the phenotypic selection process used in the breeding program before the implementation of marker-assisted selection. These genes may play a complementary role to *BPH 3* in the BPH resistance mechanisms of IL rice plants. In this study, Os04g27430 was identified as a BPH feeding-inducible *STPS* that may be involved in the BPH resistance mechanism of RH. This gene may contribute in the antixenosis mechanism by interfering with BPH settling.

## Electronic supplementary material

Additional file 1:**Data 1A: Predicted SFP called at different threshold (delta) between KDML105 and the pool of 4 introgression lines.** Data 1B: 157 predicted SFP choose in this study. (NULL 13 KB)

Additional file 2: Table S1: SFP-containing genes. (XLS 143 KB)

Additional file 3: Table S2: Functional classification of SFP-containing genes. (XLS 140 KB)

Additional file 4: SFP validation by comparison of hybridization fold change and polymorphism detected in the probe region between KD and RH. (XLS 30 KB)

Additional file 5: Table S3:Sequence polymorphisms between KD and RH in predicted SFP. (XLS 32 KB)

Below are the links to the authors’ original submitted files for images.

Authors’ original file for figure 1

Authors’ original file for figure 2

Authors’ original file for figure 3

Authors’ original file for figure 4

Authors’ original file for figure 5

Authors’ original file for figure 6

Authors’ original file for figure 7

Authors’ original file for figure 8

Authors’ original file for figure 9

Authors’ original file for figure 10

Authors’ original file for figure 11

Authors’ original file for figure 12

Authors’ original file for figure 13

Authors’ original file for figure 14

Authors’ original file for figure 15

## Abbreviations

BPH: Brown planthopper
IL: Isogenic line
KDML105: KD, Khao Dowk Mali 105
RH: Rathu Heenati
STPS: Sesquiterpene synthase
SFP: Single feature polymorphism
SNP: Single nucleotide polymorphism
TPS: Terpene synthase.
