# Supplementary material for: Single-feature polymorphism mapping of isogenic rice lines identifies the influence of terpene synthase on brown planthopper feeding preferences
Source: Rice (N Y). 2013 Aug 2;6:18. doi: 10.1186/1939-8433-6-18 (PMC4883687; doi:10.1186/1939-8433-6-18)
Supplement: Supplementary file 6 — Authors’ original file for figure 1 [file 12284_2013_58_MOESM6_ESM.pdf]

A

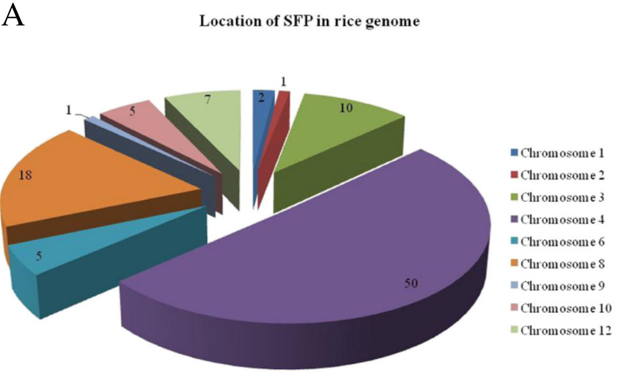

C

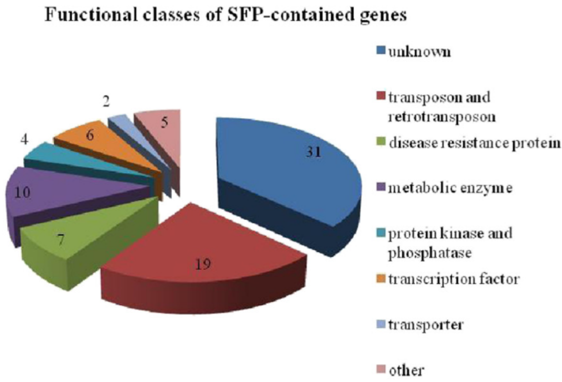

B

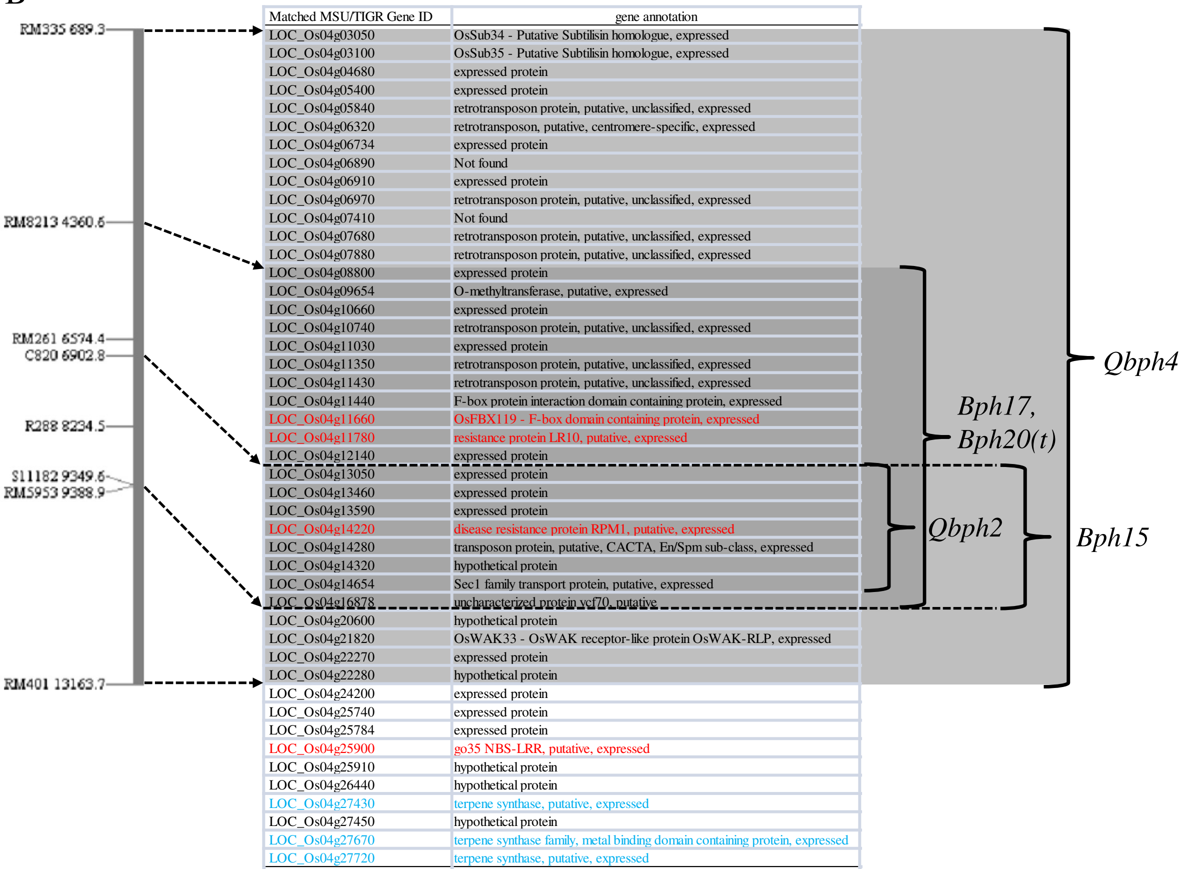

Matched MSU/TIGR Gene ID	gene annotation
LOC_Os04g03050	OsSub34 - Putative Subtilisin homologue, expressed
LOC_Os04g03100	OsSub35 - Putative Subtilisin homologue, expressed
LOC_Os04g04680	expressed protein
LOC_Os04g05400	expressed protein
LOC_Os04g05840	retrotransposon protein, putative, unclassified, expressed
LOC_Os04g06320	retrotransposon, putative, centromere-specific, expressed
LOC_Os04g06734	expressed protein
LOC_Os04g06890	Not found
LOC_Os04g06910	expressed protein
LOC_Os04g06970	retrotransposon protein, putative, unclassified, expressed
LOC_Os04g07410	Not found
LOC_Os04g07680	retrotransposon protein, putative, unclassified, expressed
LOC_Os04g07880	retrotransposon protein, putative, unclassified, expressed
LOC_Os04g08800	expressed protein
LOC_Os04g09654	O-methyltransferase, putative, expressed
LOC_Os04g10660	expressed protein
LOC_Os04g10740	retrotransposon protein, putative, unclassified, expressed
LOC_Os04g11030	expressed protein
LOC_Os04g11350	retrotransposon protein, putative, unclassified, expressed
LOC_Os04g11430	retrotransposon protein, putative, unclassified, expressed
LOC_Os04g11440	F-box protein interaction domain containing protein, expressed
LOC_Os04g11660	OsFBX119 - F-box domain containing protein, expressed
LOC_Os04g11780	resistance protein LR10, putative, expressed
LOC_Os04g12140	expressed protein
LOC_Os04g13050	expressed protein
LOC_Os04g13460	expressed protein
LOC_Os04g13590	expressed protein
LOC_Os04g14220	disease resistance protein RPM1, putative, expressed
LOC_Os04g14280	transposon protein, putative, CACTA, En/Spm sub-class, expressed
LOC_Os04g14320	hypothetical protein
LOC_Os04g14654	Sec1 family transport protein, putative, expressed
LOC_Os04g16878	uncharacterized protein ycf70, putative
LOC_Os04g20600	hypothetical protein
LOC_Os04g21820	OsWAK33 - OsWAK receptor-like protein OsWAK-RLP, expressed
LOC_Os04g22270	expressed protein
LOC_Os04g22280	hypothetical protein
LOC_Os04g24200	expressed protein
LOC_Os04g25740	expressed protein
LOC_Os04g25784	expressed protein
LOC_Os04g25900	go35 NBS-LRR, putative, expressed
LOC_Os04g25910	hypothetical protein
LOC_Os04g26440	hypothetical protein
LOC_Os04g27430	terpene synthase, putative, expressed
LOC_Os04g27450	hypothetical protein
LOC_Os04g27670	terpene synthase family, metal binding domain containing protein, expressed
LOC_Os04g27720	terpene synthase, putative, expressed
