# Supplementary figures and images for: Single-feature polymorphism mapping of isogenic rice lines identifies the influence of terpene synthase on brown planthopper feeding preferences
Source: Rice (N Y). 2013 Aug 2;6:18. doi: 10.1186/1939-8433-6-18 (PMC4883687; doi:10.1186/1939-8433-6-18)

**A**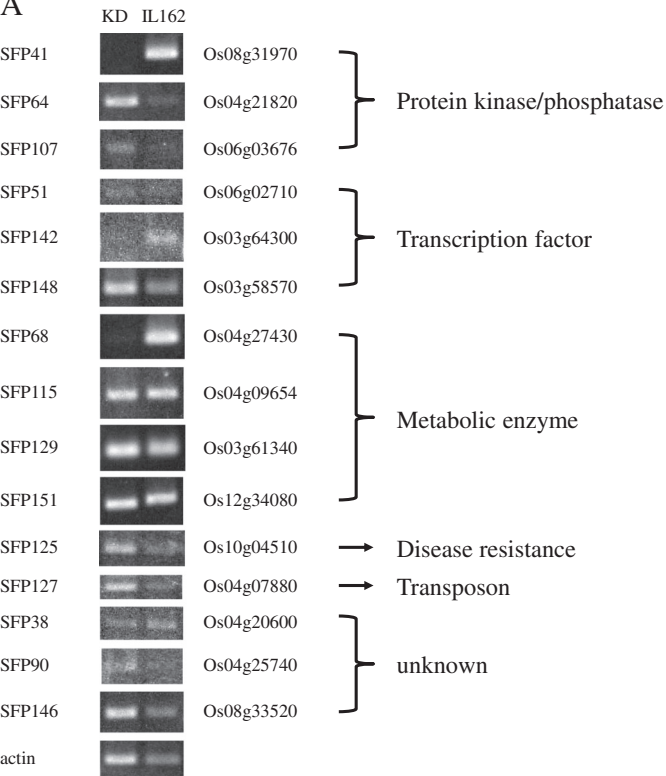**B**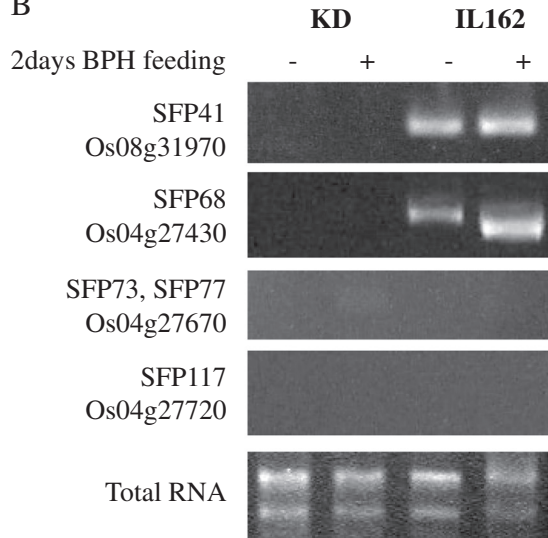

Supplement: Supplementary file 7 — Authors’ original file for figure 2 [file 12284_2013_58_MOESM7_ESM.pdf]

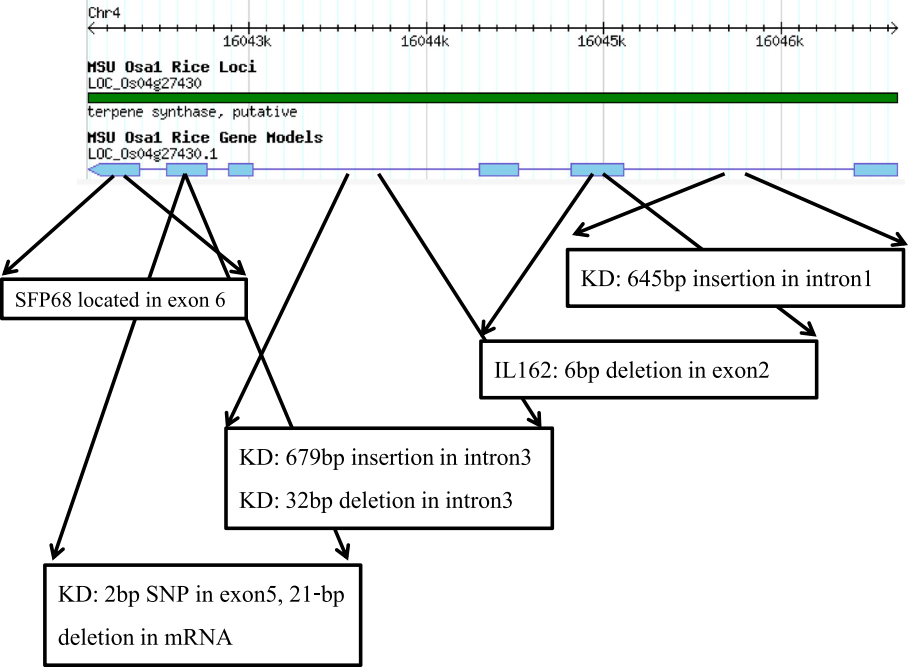

Supplement: Supplementary file 8 — Authors’ original file for figure 3 [file 12284_2013_58_MOESM8_ESM.pdf]

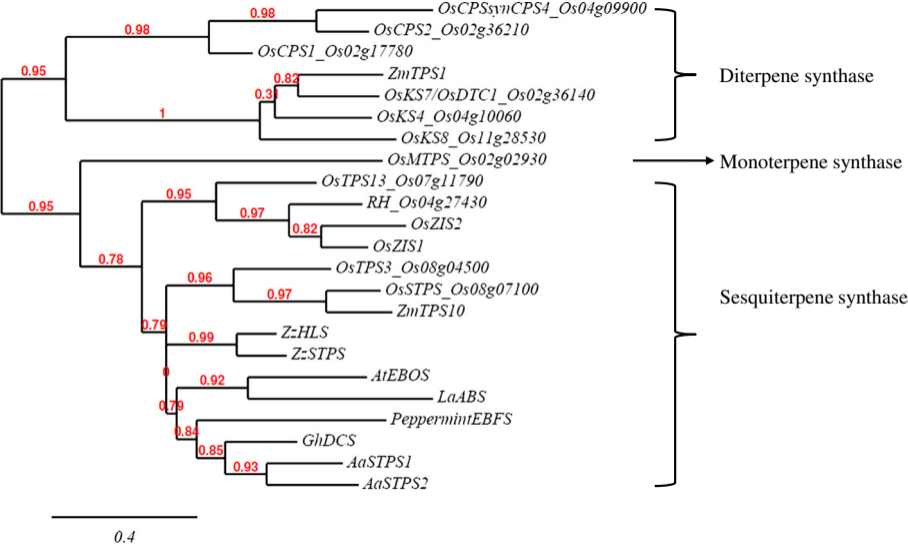

Supplement: Supplementary file 11 — Authors’ original file for figure 6 [file 12284_2013_58_MOESM11_ESM.pdf]

**A**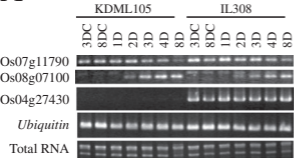**B**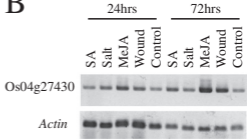

Supplement: Supplementary file 12 — Authors’ original file for figure 7 [file 12284_2013_58_MOESM12_ESM.pdf]

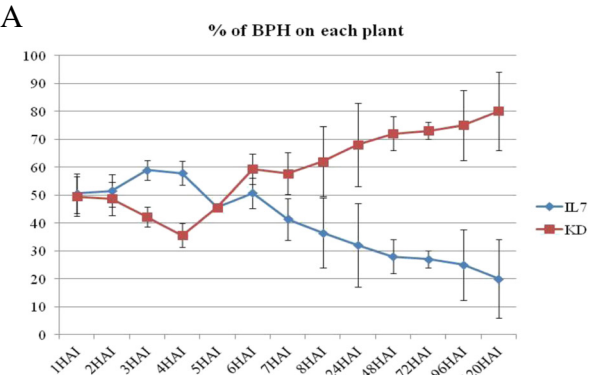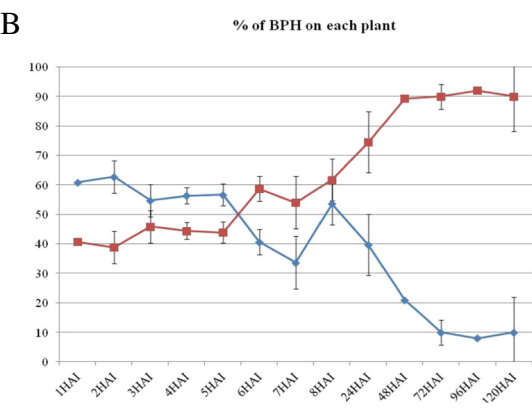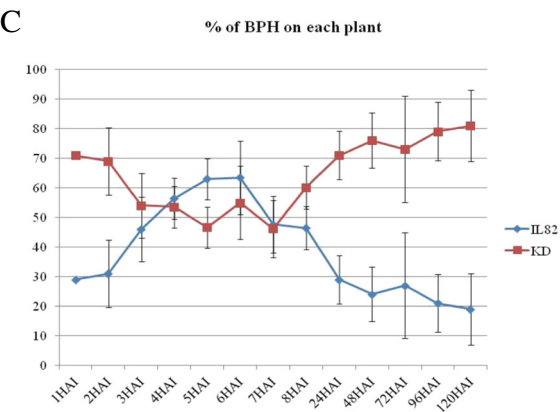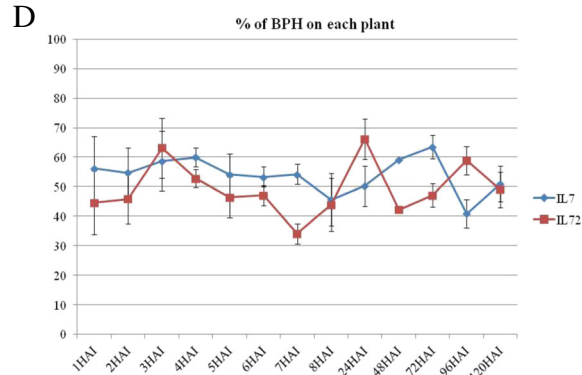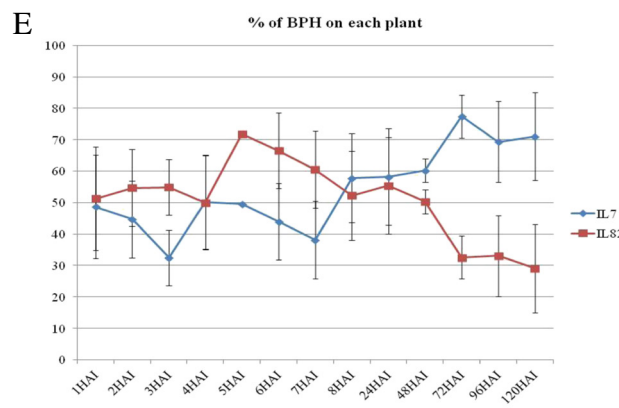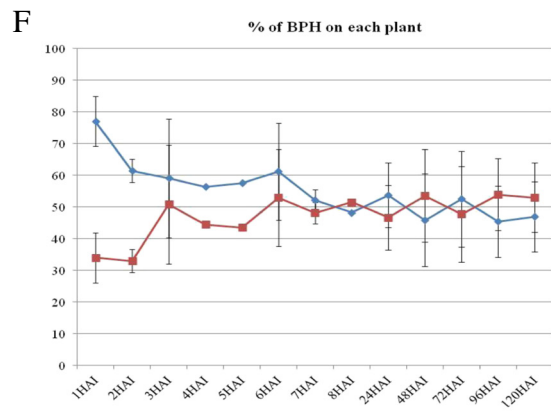

Supplement: Supplementary file 13 — Authors’ original file for figure 8 [file 12284_2013_58_MOESM13_ESM.pdf]

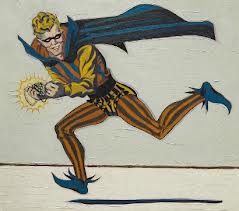

Supplement: Supplementary file 14 — Authors’ original file for figure 9 [file 12284_2013_58_MOESM14_ESM.jpeg]

## Slide 1
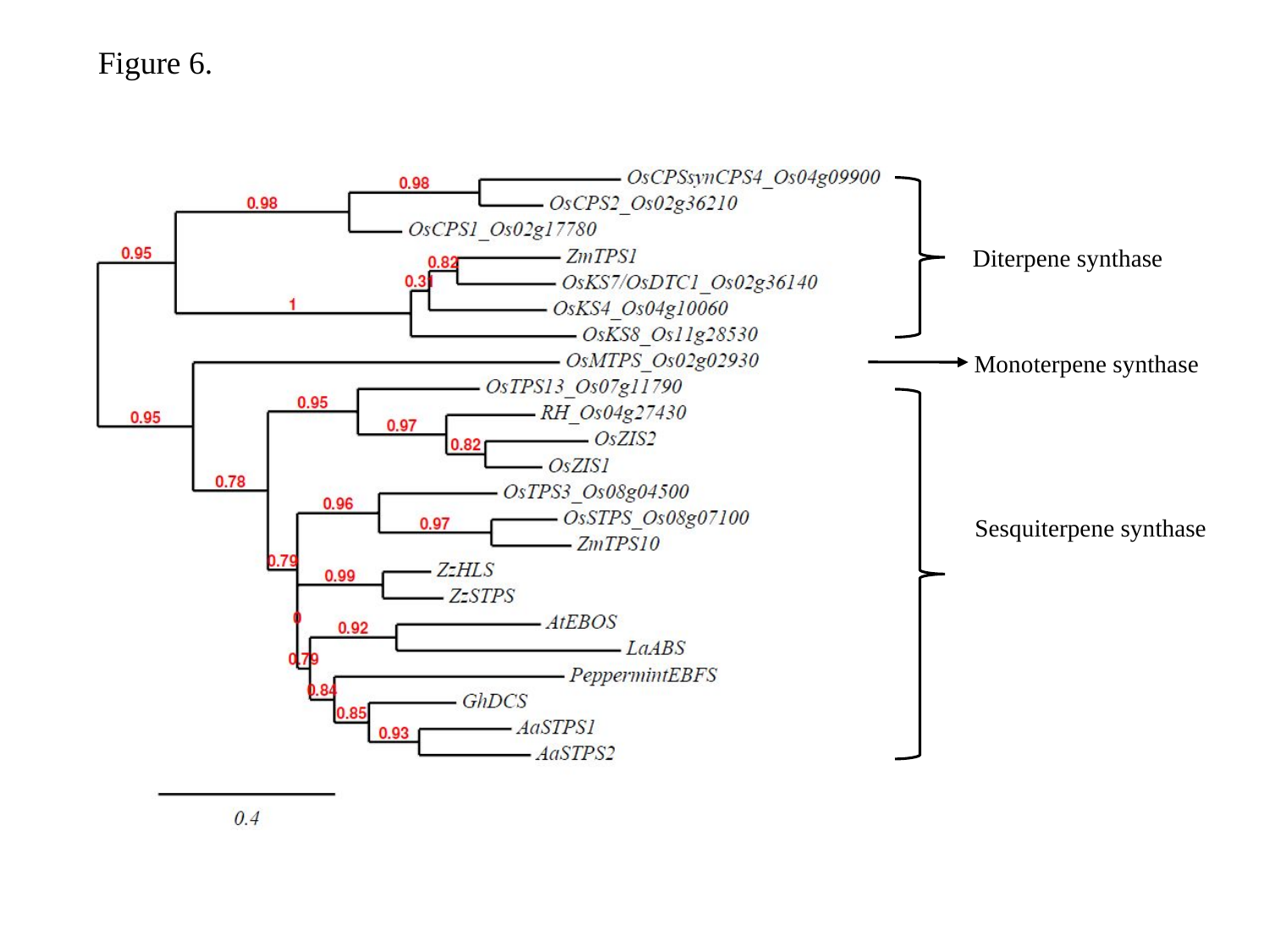

Figure 6.
Diterpene synthase
Monoterpene synthase
Sesquiterpene synthase

Supplement: Supplementary file 15 — Authors’ original file for figure 10 [file 12284_2013_58_MOESM15_ESM.ppt]

## Slide 1
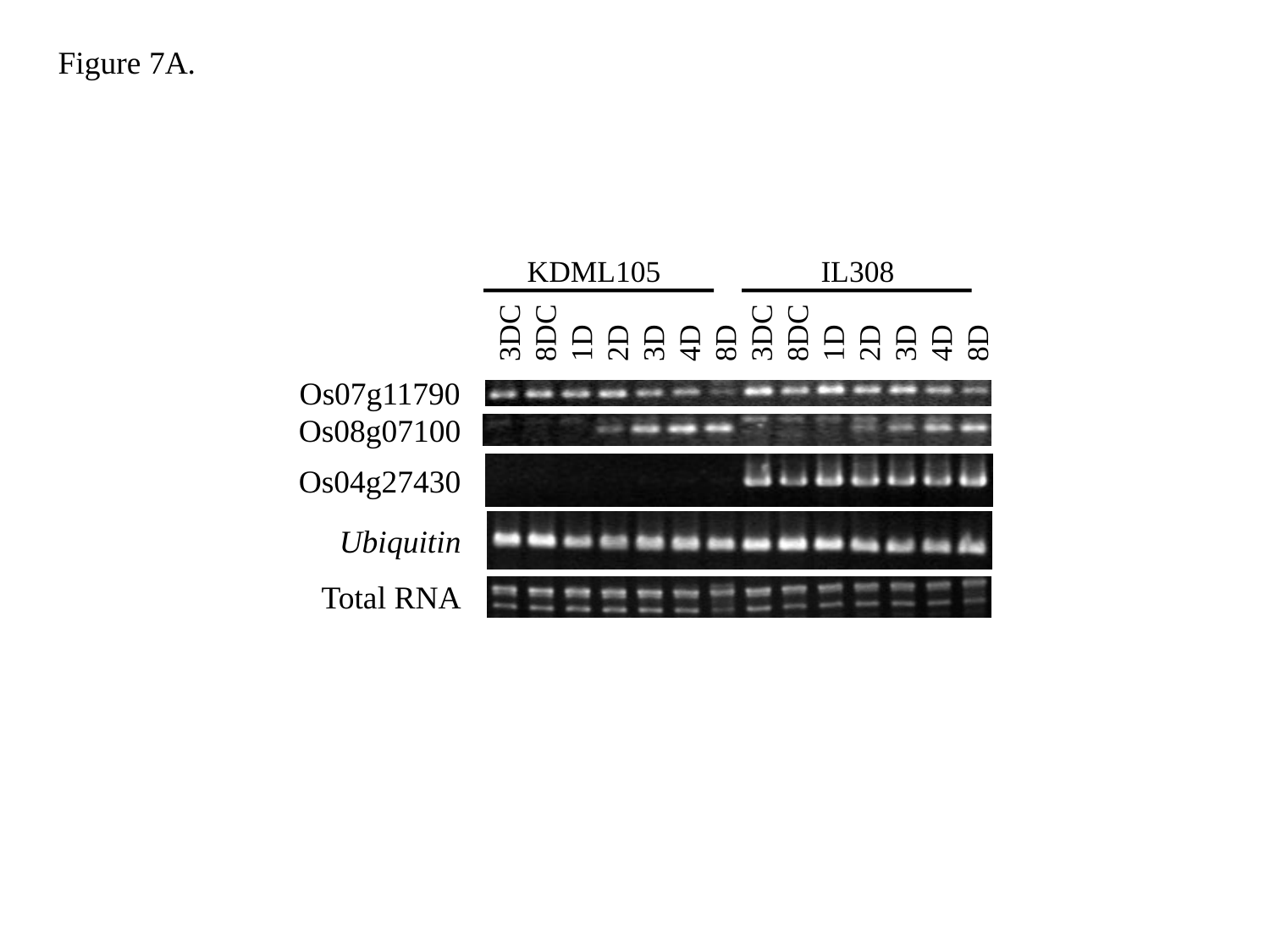

Figure 7A.
3DC
8DC
1D
2D
3D
4D
8D
3DC
8DC
1D
2D
3D
4D
8D
KDML105
IL308
Os07g11790
Os08g07100
Os04g27430
Ubiquitin
Total RNA

Supplement: Supplementary file 16 — Authors’ original file for figure 11 [file 12284_2013_58_MOESM16_ESM.ppt]

## Slide 1
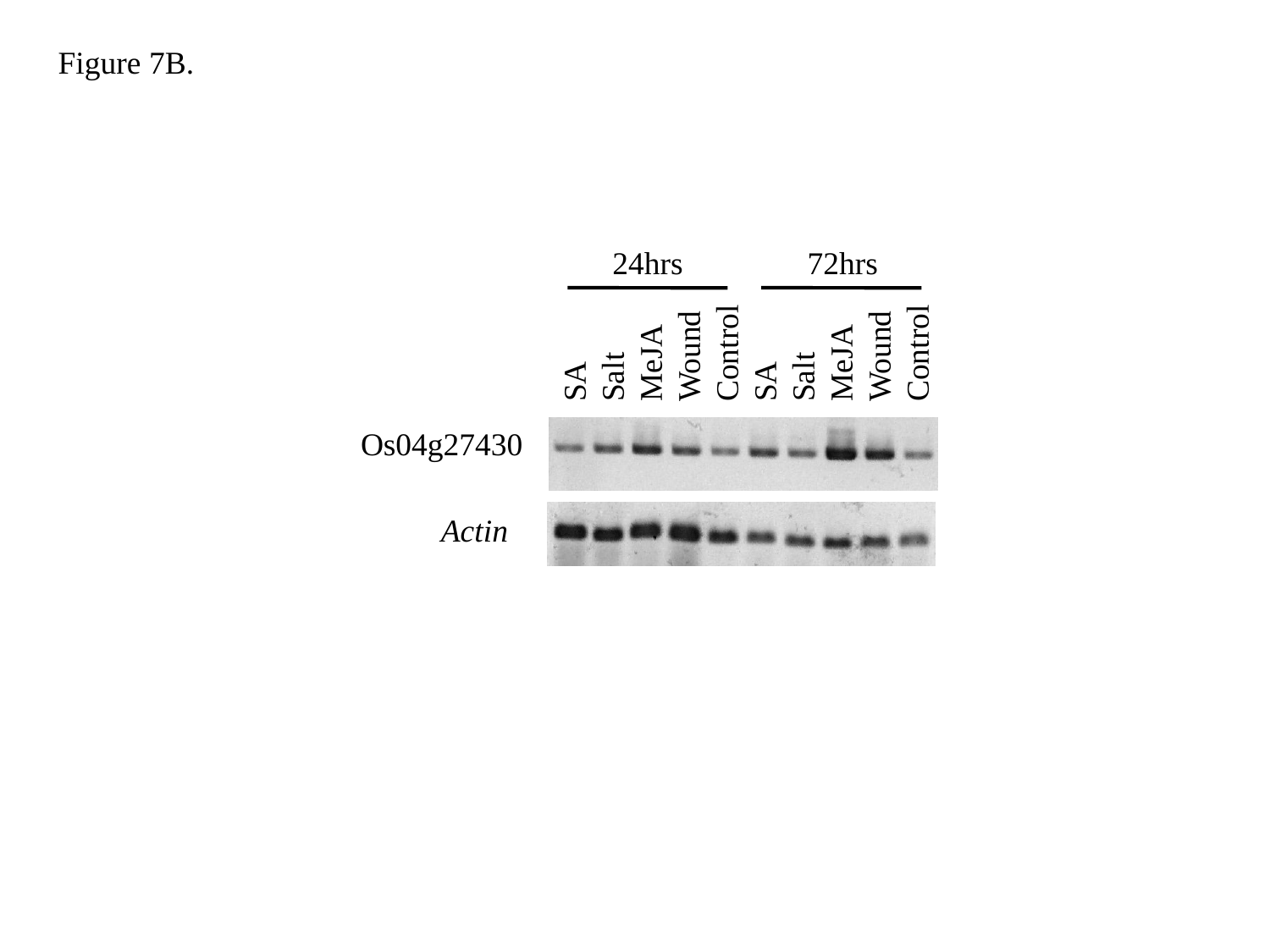

Figure 7B.
SA
Salt
MeJA
Wound
Control
SA
Salt
MeJA
Wound
Control
24hrs
72hrs
Os04g27430
Actin

Supplement: Supplementary file 17 — Authors’ original file for figure 12 [file 12284_2013_58_MOESM17_ESM.ppt]

## Slide 1
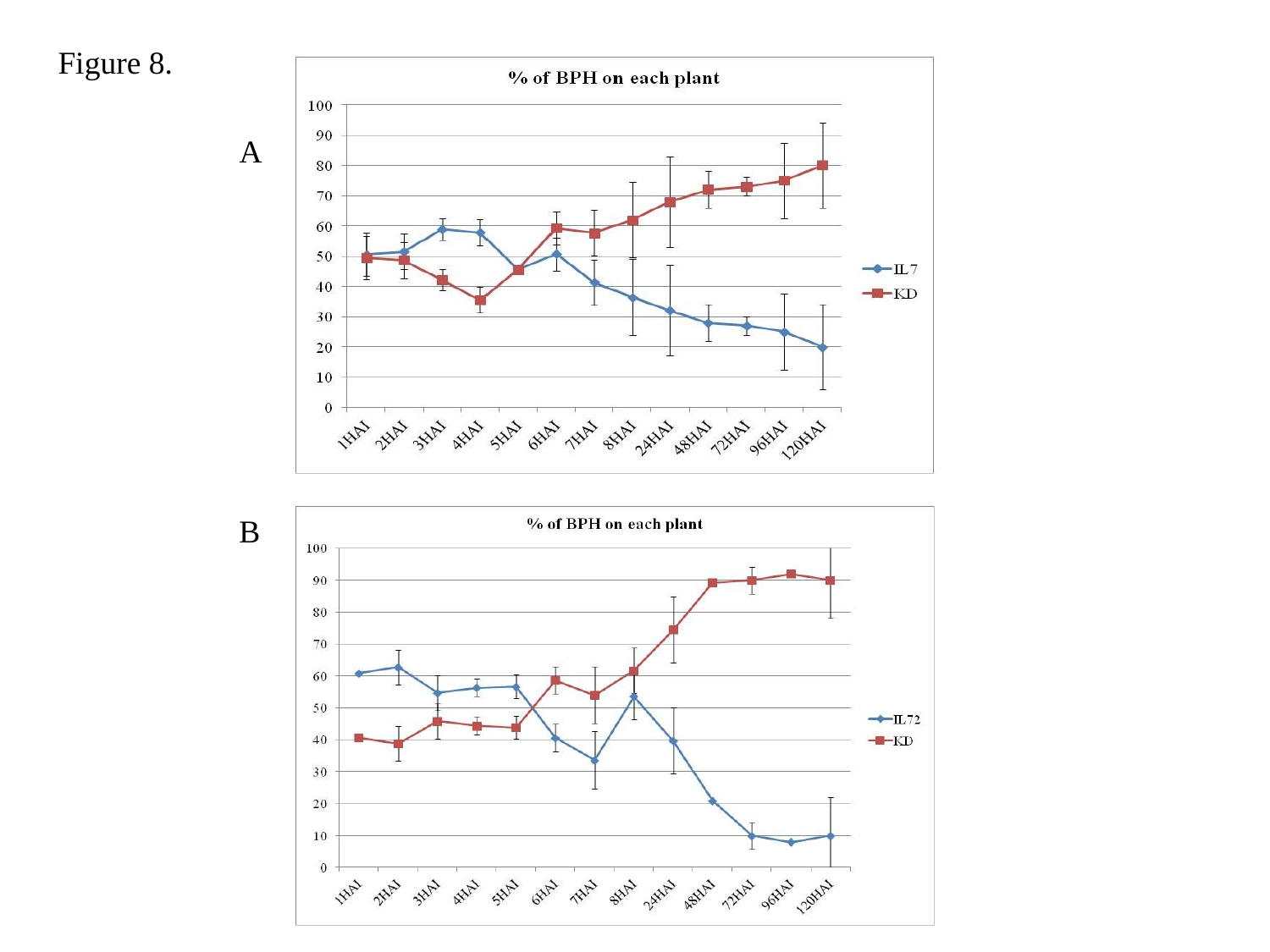

Figure 8.
A
B

Supplement: Supplementary file 18 — Authors’ original file for figure 13 [file 12284_2013_58_MOESM18_ESM.ppt]

## Slide 1
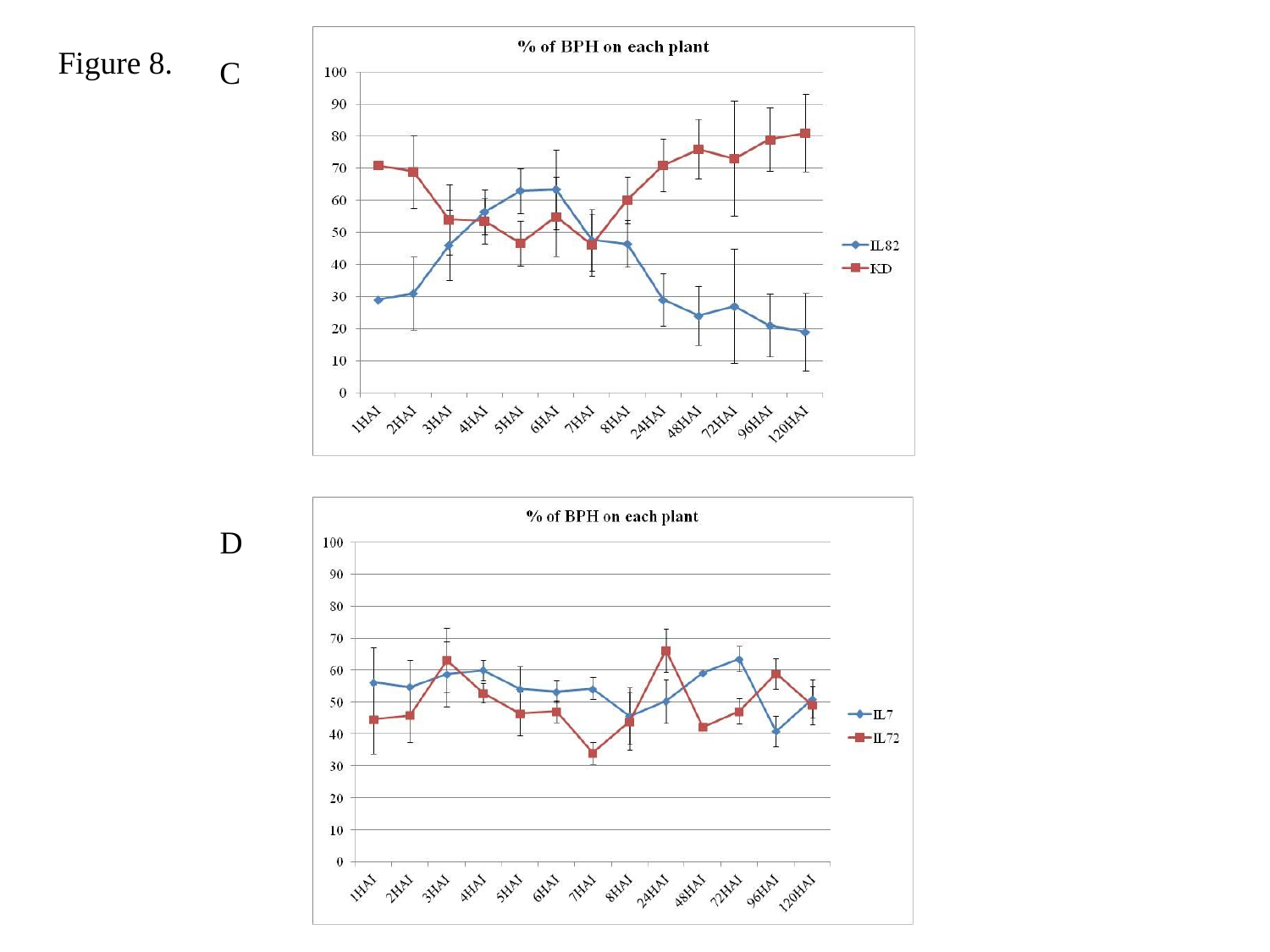

Figure 8.
C
D

Supplement: Supplementary file 19 — Authors’ original file for figure 14 [file 12284_2013_58_MOESM19_ESM.ppt]

## Slide 1
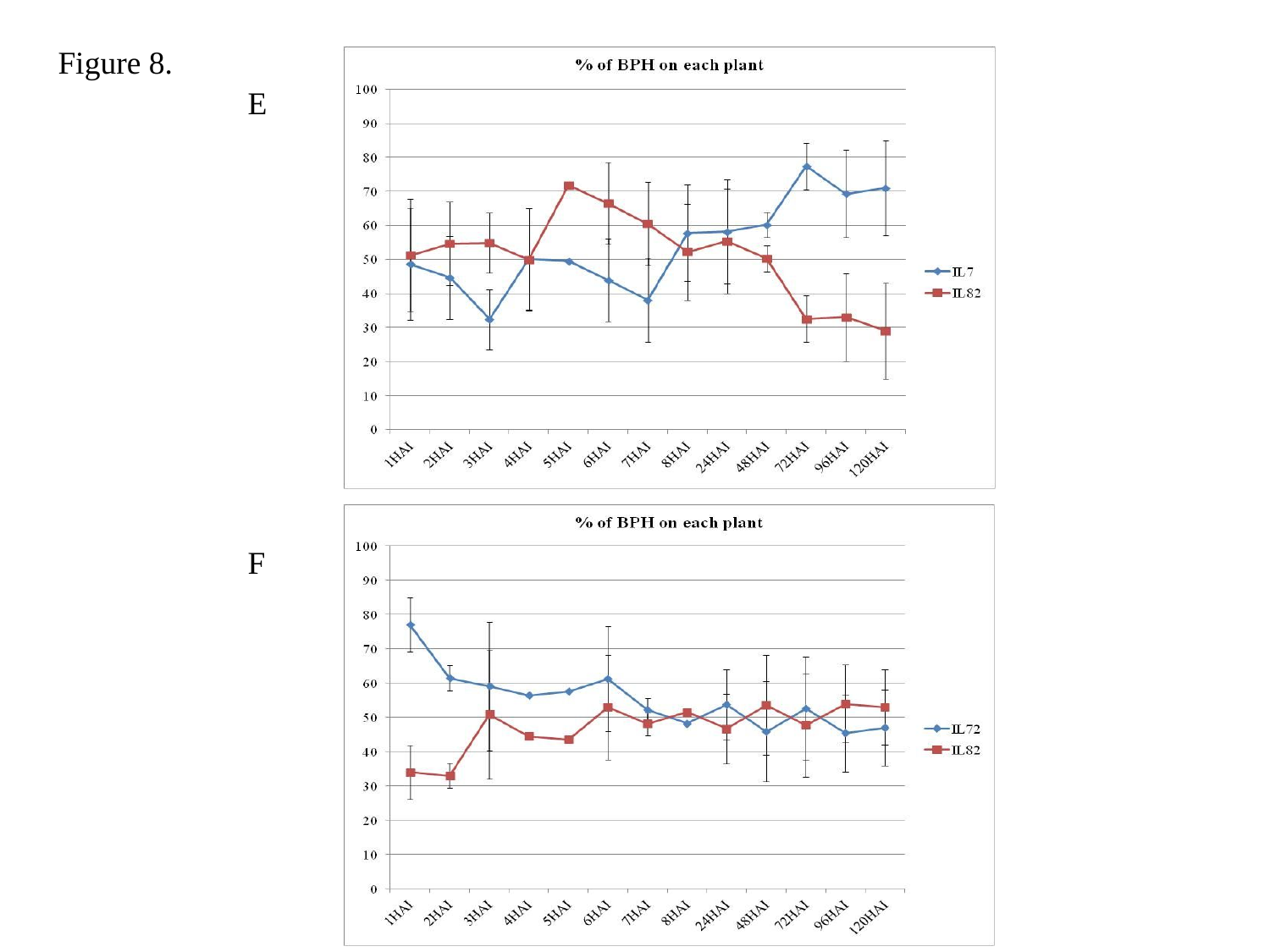

Figure 8.
E
F

Supplement: Supplementary file 20 — Authors’ original file for figure 15 [file 12284_2013_58_MOESM20_ESM.ppt]
