# Supplementary material for: Single-feature polymorphism mapping of isogenic rice lines identifies the influence of terpene synthase on brown planthopper feeding preferences
Source: Rice (N Y). 2013 Aug 2;6:18. doi: 10.1186/1939-8433-6-18 (PMC4883687; doi:10.1186/1939-8433-6-18)
Supplement: Supplementary file 9 — Authors’ original file for figure 4 [file 12284_2013_58_MOESM9_ESM.pdf]

A

```
RH          MRKRADELKEKVRTQLGTCEDIVGTMNLVDAIQHLGIEHLFKQEIDNTLRDIRTSEFTSS 60
IL162       MRKRADELKEKVRTQLGTCEDIVGTMNLVDAIQHLGIEHLFKQEIDNTLRDIRTSEFTSS 60
KD          MRKRADELKEKVRTQLGTCEDIVGTMNLVDAIQHLGIEHLFKQEIDNTLRDIRTSEFTSS 60
            ************************************************************

RH          SLHEVALWFRLLREHGLWVSPDVFG--KFDGDDARLSSVIADHDTRGLLSLYNAAHLLVH 118
IL162       SLHEVALWFRLLREHGLWVSPDVFG--KFDGDDARLSSVIADHDTRGLLSLYNAAHLLVH 118
KD          SLHEVALWFRLLREHGLWVSPDVFGKFKFDGDDARLSSVIADHDTRGLLSLYNAAHLLVH 120
            *************************  *********************************

RH          GEPELEEAISIARHHLKSMTRDCDLNPVLANQVNRALNIALPRTCKRLETSLFISEYEQE 178
IL162       GEPELEEAISIARHHLKSMTRDCDLNPVLANQVNRALNIALPRTCKRLETSLFISEYEQE 178
KD          GEPELEEAISIARHRLKSMTRDCDLNPVLANQVNRALNIALPRTCKRLETSLFISEYEQE 180
            **************:*********************************************

RH          EGYSEILLELAKLDFNIVQNVHLMELKSISEWWRDLYTYVGLNYARDRAVEGYLWSCLVF 238
IL162       EGYSEILLELAKLDFNIVQNVHLMELKSISEWWRDLYTYVGLNYARDRAVEGYLWSCLVF 238
KD          EGYSEILLELAKLDFNIVQNVHLMELKSISEWWRDLYTYVGLNYARDRAVEGYLWSCLVF 240
            ************************************************************

RH          YEKDLSFTRTFVAKMILLVTLMDDTFDSHATIQECRQLNSAIQRWDESAVTLLPEYLKKF 298
IL162       YEKDLSFTRTFVAKMILLVTLMDDTFDSHATIQECRQLNSAIQRWDESAVTLLPEYLKKF 298
KD          YEKDLSFTRTFVAKMILLVTLMDDTFDSHATIQECRQLNSAIQRWDESAVTLLPEYLKKF 300
            ************************************************************

RH          YRELLRNFKVLQDQVTDNDKYRVTYTRKEFQKLSTYYLQEAEWSHQRHKPSFGDQITLTA 358
IL162       YRELLRNFKVLQDQVTDNDKYRVTYTRKEFQKLSTYYLQEAEWSHQRHKPSFGDQITLTA 358
KD          YRELLRNFKVLQDQVTDNDKYRVTYTRKEFQKLSTYYLQEAE-------PSFGDQITLTA 353
            ******************************************       ***********

RH          MSSVIPLLCVSGTVGMGYVTKETFEWVASRTTAIVASAKIGRFMNDIAAMKRGKNKGDVA 418
IL162       MSSVIPLLCVSGTVGMGYVTKETFEWVASRTTAIVASAKIGRFMNDIAAMKRGKNKGDVA 418
KD          MSSVIPLLCVSGTVGMGYVTMETFEWVASRTTAIVASAKIGRFMNDIAAMKRGKNKGDVA 413
            ******************** ***************************************

RH          SSVECYMNEHKVTMEVAIDKIDSLVEDEWRTLNQAHFEDHKLFPVVEQVVNLTASMASFY 478
IL162       SSVECYMNEHKVTMEVAIDKIDSLVEDEWRTLNQAHFEDHKLFPVVEQVVNLTASMASFY 478
KD          SSVECYMNEHKVTMEVAIDKIDSLVEDEWRTLNQAHFEDHKLFPVVEQVVNLTASMASFY 473
            ************************************************************

RH          DERKDAYTFPTLLQDTIESLFVNPVPI- 505
IL162       DERKDAYTFPTLLQDTIESLFVNPVPI- 505
KD          DERKDAYTFPTLLQDTIESLFVNPVPI- 500
            ***************************
```

B

```
OsKS4_diTPS      dvtkYnveswldylrslatdAEWqrskYvPtmEeymknSiVtfaLgptiliAlyfMGq
MonoTPS          npInhlKKawamLfdgfMtEtkWlsaGqvPdsEeyLrngvVtSgVPLvfVhllfmLGh
ZmTPS8           YRVaYarKaYQlLSksYLQEvEWcHQGYtPSFDDhVSVStaSAgIqvLCVgmlVGMGD
OsZIS1           YRVTcmKKEFQnLSTYYLQEfEWlHQnYkPaFKerVALStLSStVPLLCatAaVGqGD
RH_Os04g27430    YRVTYTrKEFQkLSTYYLQEAEWsHQrhkPSFgDQITLtAMSSvIPLLCVsgtVGMGy
KD_Os04g27430    YRVTYTrKEFQkLSTYYLQEAE-------PSFgDQITLtAMSSvIPLLCVsgtVGMGy
Nip_Os04g27430   YRVTYTrKEFQkLSTYYLQEAE-------PSFgDQITLtAMSSvIPLLCVsgtVGMGy
AaEBFS           YQIhYvKemakeLvrnYLvEArWlkEGYmPtlEeymSVSmVtgtygLMiarSyVGrGD
CjEBFS           YgIpYaKqmmQeLiilYftEAkWlYkGYvPtFDeykSValrSigLrtLaVaSfVdLGD
```
