# Supplementary material for: Single-feature polymorphism mapping of isogenic rice lines identifies the influence of terpene synthase on brown planthopper feeding preferences
Source: Rice (N Y). 2013 Aug 2;6:18. doi: 10.1186/1939-8433-6-18 (PMC4883687; doi:10.1186/1939-8433-6-18)
Supplement: Supplementary file 10 — Authors’ original file for figure 5 [file 12284_2013_58_MOESM10_ESM.pdf]

```
RH      TTTCAGCAATTCTATATCTTTTATAATGTAGGTAGGGTACGTATATCATAGTTTTATTAT 60
IL162   TTTCAGCAATTCTATATCTTTTATAATGTAGGTAGGGTACGTATATCATAGTTTTATTAT 60
KD      TTTCAGCAATTCTATATCGTTTATAATGTAGGTAGGGTACGTATATCATAGTTTTATTAT 60
        ****************** *****************************************

RH      TTGCGTAGCCTAAATATTTGATGGACTTCCTACGTGATGAGATGATCACAAAAATTAACA 120
IL162   TTGCGTAGCCTAAATATTTGATGGACTTCCTACGTGATGAGATGATCACAAAAATTAACA 120
KD      TTGCGTAGCCTAAATATTTGATGGACTTCCTACGTGATGAGATGATCACAAAAATTAACA 120
        ************************************************************

RH      ATCAACATGTTCAGATTTGAATAAGCGCTTATATTCAAGCAGAATTAATGTGTACATATA 180
IL162   ATCAACATGTTCAGATTTGAATAAGCGCTTATATTCAAGCAGAATTAATGTGTACATATA 180
KD      ATCAACATGTTCAGATTTGAATAAGCGCTTATATTCAAGCAGAATTAATGTGTACATATA 180
        ************************************************************

RH      TTTCTGATGTTTTTCTTGCACATGGTATTAGTTCATGTAAGATTATCTTTCCTTTTTTTT 240
IL162   TTTCTGATGTTTTTCTTGCACATGGTATTAGTTCATGTAAGATTATCTTTCCTTTTTTTT 240
KD      TTTCTGATGTTTTTCTTGCACATGGTATTAGTTCATGTAAGATTATCTTTCCTTTTTTTT 240
        ************************************************************     TF search
                                                                           score
                                   ATHB-1
RH      CATAATTTCTGCATGTACTACTTTTGACAAATTAATTATTGCTAGAACTGTTTCATGAAT 300    87.2
IL162   CATAATTTCTGCATGTACTACTTTTGACAAATTAATTATTGCTAGAACTGTTTCATGAAT 300    87.2
KD      CATAATTTCTGCATGTACTACTTTTGACAAATTCATTATTGCTAGAACTGTTTCATGAAT 300    0
        *********************************.**************************

                                     SBF-1
RH      ATTGTAATGTTATTTCACAAAGCTTTGTGTGGTTAATAATCTCTTTTTGATAACTATAAT 360    98.1
IL162   ATTGTAATGTTATTTCACAAAGCTTTGTGTGGTTAATAATCTCTTTTTGATAACTATAAT 360    98.1
KD      ATTGTAATGTTATTTCACAAAGCTTTGCATGGTTAATAATCTCTTTTTGATAACTATAAT 360    92.5
        *************************** .*******************************

                               P
RH      AAATCAAACTCGTGAATCCAACCAACCAGAGATCGGAAGAATGG 404                    98.2
IL162   AAATCAAACTCGTGAATCCAACCAACCAGAGATCGGAAGAATGG 404                    98.2
KD      AAATCAAACTCGTGAATCCAACTAACCAGAGATCGGAAGAATGG 404                    86.5
        ********************** *********************
```
